# Diagnostic Performance of Dengue Virus Envelope Domain III in Acute Dengue Infection

**DOI:** 10.3390/ijms20143464

**Published:** 2019-07-15

**Authors:** Ngoc Minh Nguyen, Bao Tuan Duong, Mudsser Azam, Truong Thai Phuong, Hyun Park, Phung Thi Bich Thuy, Seon-Ju Yeo

**Affiliations:** 1 Zoonosis Research Center, Department of Infection Biology, School of Medicine, Wonkwang University, Iksan 570–749, Korea; 2 Department of Microbiology, Bach Mai Hospital, Hanoi 100000, Vietnam; 3 Department of Research of Biomolecular for Infectious Disease, National Children’s Hospital, Hanoi 100000, Vietnam

**Keywords:** dengue virus recombinant envelope protein domain III, detection of IgM of dengue patient, acute phase infection

## Abstract

Dengue, one of the most prevalent illnesses caused by dengue viruses that are members of the genus *Flavivirus*, is a significant global health problem. However, similar clinical symptoms and high antigenic homologies with other Flaviviruses in the endemic area pose difficulties for differential diagnosis of dengue from other arbovirus infections. Here, we investigated four types of recombinant envelope protein domain III (DV-rED III) derived from four dengue virus (DENV) serotypes for diagnostic potential in detecting IgM in acute phase (mainly 2–3 days after onset of fever). Each independent DV-1, -3, and -4-rED III-ELISA showed less than 60% sensitivity, but the combined results of DV-1, -3, and -4-rED III-ELISA led to sensitivity of 81.82% (18/22) (95% CI, 59.72 to 94.81) and 100% specificity (46/46) (95% CI, 92.29 to 100.00) as each antigen compensated the other antigen-derived negative result. In conclusion, the independent combination of data derived from each recombinant antigen (DV1-, DV3-, and DV4-rED III) showed comparable efficacy for the detection of IgM in patients with acute-phase dengue infection.

## 1. Introduction

Dengue disease is a global public health threat caused by the spread of four distinct serotypes of dengue virus (DENV-1, -2, -3, and -4), with the prevalence of the disease having dramatically increased in recent decades [1]. Dengue disease can vary from mild dengue fever to severe, life-threatening syndromes, dengue hemorrhagic fever (DHF), and dengue shock syndrome (DSS) [2]. The increasing trend in the incidence of dengue infection is of great concern, as there is no specific treatment for dengue, and most forms of therapy are supportive in nature because vaccine is as yet unavailable [3]. Although a primary infection confers lifelong protective immunity against the infecting serotype, cross-protection against any of the other three serotypes during secondary infection with a heterologous serotype poses a risk for DHF and DSS [4]. This necessitates early detection of disease to assist with the clinical management of symptoms. However, 70% of all dengue-infected individuals are asymptomatic, causing a problem of blood transmission in all tropical and subtropical countries, which are dengue-endemic areas [5,6,7,8].

Isolation of the virus and the detection of viral RNA using RT-PCR are commonly used methods for early diagnosis but are time-consuming, expensive and require skilled operation.

Additionally, the DENV genome can only be detected in the blood (serum) of patients for approximately the first 5 days of symptoms [9]. The window for viral RNA detection is limited to 5 to 7 days after initial infection, and patients often arrive at the hospital too late for viral RNA detection [10]. In addition, serological diagnostics require the patient to return in 7 to 10 days [10].

The urgent need for rapid, accurate and affordable diagnostics has been accelerated with WHO declaring the Zika outbreak a health emergency of international concern. Since dengue, Zika, and chikungunya all display similar symptoms, developing tests that can be used outside of a laboratory and are sufficiently sensitive to differentiate each of these diseases is a top priority [11].

The DENV genome is translated as a single polypeptide, which is then cleaved by viral and cellular proteases into three structural proteins (C, prM/M, E) and seven nonstructural proteins (NS1, NS2A, NS2B, NS3, NS4A, NS4B, NS5) [12]. NS1 is a highly conserved glycoprotein that is present at high concentrations in sera of dengue-infected patients during the early clinical phase of the disease, and found from Day 1 to Day 9 after onset of fever in primary or secondary dengue-infected patients [13]. However, cross-reactivity between flaviviruses has been reported in antibody assays and in tests for dengue (NS1) antigen, although recently, a pair of antibodies in rapid immunochromatography tests showed specificity without cross reaction between Zika and DENV [14,15].

Alternatively, envelope (E) protein is responsible for eliciting a neutralizing antibody response via domain III [16] and thus, it has been used for serological diagnosis of dengue by determination of IgM. In contrast to IgG, which appears by the 14th day and persists for life, DENV IgM reaches detectable levels in nearly all DENV-infected patients within 5 days of symptom onset and reaches peak levels approximately 2 weeks later. Therefore, detection of IgM is important to treat patients during acute phase of the disease [17,18]. Immunoglobulin responses to DENV exhibit significant levels of IgM to each domain of envelope (E) protein, indicating that E protein domain III cross-reactive immunoglobulin populations were similarly variable and much larger in IgM than in IgG [19]. A DENV-2 envelope domain III protein has been evaluated as a vaccine target [20,21], but it showed limitation for discrimination of ZIKV due to broad cross-reactivity [22], although ZIKV envelope protein domain III was reported to have high specificity to determine ZIKV infection from DENV by detection of IgG [23]. The use of DENV-1 envelope protein domain III expressed in methylotrophic yeast *Pichia pastoris* to develop dengue-specific IgM detection (114 positive and 46 negative) has been reported [24].

In this study, we expressed DENV envelope domain III from all four serotypes in *Escherichia coli* and evaluated their efficacy to detect dengue-specific IgM levels in infected patients.

## 2. Results

### 2.1. Expression of Recombinant Envelope Domain III Proteins

Four dengue viruses (DV1, DV2, DV3, and DV4) were successfully amplified and titrated (Figure S1).

**Figure 1:**
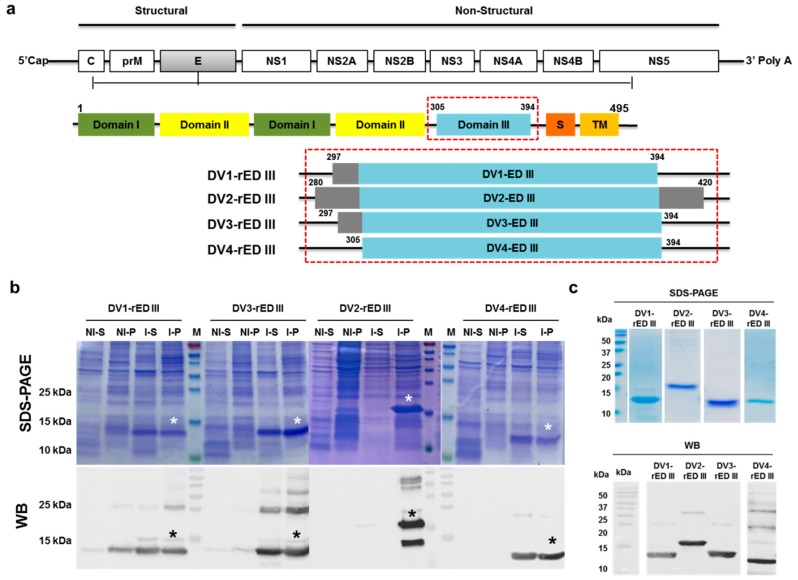
Expression and purification of recombinant DENV envelope domain III. (**a**) Domain III of envelope protein from each DENV serotype was amplified by PCR and cloned in pET21b. DV1-rED III, DENV-1 envelope domain III (297–394 amino acids (a.a.)); DV2-rED III, DENV-2 envelope domain III (280–420 a.a.); DV3-rED III, DENV-3 envelope domain III (297–394 a.a.); DV4-rED III, DENV-4 envelope domain III (305–394 a.a.). (**b**) The recombinant antigens were induced by IPTG and the presence of antigens in pellet and supernatants was confirmed by SDS-PAGE and western blotting (WB), using anti-6×His tag. (**c**) The purified antigens were confirmed by SDS-PAGE and WB. Asterisks indicate the expressed and used antigen fraction for further study. NI-S, non-induced soluble fraction; NI-P, non-induced pellet; I-S, induced soluble fraction; I-P, induced pellet; M, marker.

The respective gene fragments for developing recombinant diagnostic antigens of DV1-, DV2-, DV3-, DV4-rED III were amplified by PCR from the culture of four types of dengue viruses (Figure 1a). Since domain III of envelope protein is immunogenic [25], their amino acid homology is less than 66%, and thus, we chose domain III, derived from each type (Figure S2).

The amplified DENV envelope domain III fragments were digested with appropriate restriction enzymes, ligated into expression vector pET21b (+) to obtain a final construct. The purified recombinant antigens fused with 6×His-tag were analyzed using SDS-PAGE and western blot (Figure 1b). The recombinant DV1-, DV2-, DV3-, DV4-rED III antigens each showed a dominant band at 13, 18, 13, and 12 kDa, respectively. DV1;3;4-rED III were expressed in both soluble and pellet form after induction with IPTG, whereas DV2-rED III protein was only expressed as an insoluble fraction; therefore, all four antigens in the pellet were solubilized and refolded for further study (Figure 1c).

### 2.2. Prediction of Antigenic Epitope in Recombinant Antigens

**Figure 2:**
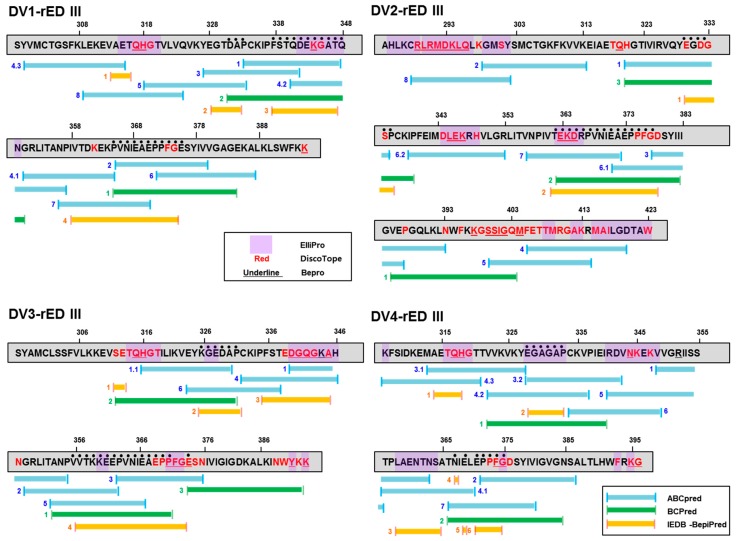
Epitope analysis of recombinant DENV envelope domain III. Each amino acid sequence from DV1-rED III to DV4-rED III was analyzed for potential linear epitope by three independent programs (ABCpred, BCPreds, and immune epitope database and analysis resource [IEDB]-BepiPred). Dots indicate the common epitope sequence found by all three programs. Additionally, three different programs (ElliPro, DiscoTope, and Bepro) were used for analysis of conformational epitopes.

To predict the potential epitope sequence in domain III, each sequence was analyzed using bioinformatics. Three independent epitope prediction programs (ABCpred (threshold: 0.7), BCPreds (specificity: 75%), and immune epitope database and analysis resource [IEDB]-BepiPred (threshold 0.7)) were used to predict the potential linear epitopes (Figure 2). ABCpred, BCPreds server 1.0, and IEDB-BepiPred predicted 21 (threshold: 0.7), 16 (specificity: 75%), and 8 (threshold 0.5) linear epitopes, respectively.

DV1-rED III showed DAP (L1-1) at 331–333, FSTQDEKGATQ (L1-2) at 337–347, and PVNIEAEPPFG (L1-3) at 365–375, as predictions by the three programs. In DV2-rED III, EGDGSP (L2-1) at 330–335 and TEKDRPVNIEAEPPFG (L2-2) at 362–377 were predicted by the three programs. DV3-rED III showed KGEDAP (L3-1) at 326–331, VVTKKEEPVNIEAEPP (L3-2) at 356–371, and E (L3-3) at 374 as common epitopes. DV4-rED III showed EGAGAP (L4-1) at 328-333, N (L4-2) at 367, E (L4-3) at 369, and EPPFG (L4-4) at 371–375 as common epitopes.

Interestingly, two epitopes (L1-1) (DAP at 331–333) and L1-3 (VNIEAEPP at 365–375) of DV1-rED III were also found in DV3-rED III as L3-1 at 326–331 and L3-2 at 356–371. DV2- and DV4-rED III showed the same epitope sequence (EPPFG at 371–375) as L2-2 at 362–377 and L4-4 at 371–375, respectively.

DV1-rED III showed two conformational epitopes with QH (C1-1) at 317–318 and K (C1-2) at 344. DV2-rED III presented three epitopes with RLRMDKLQ (C2-1) at 289–396, LEK (C2-2) at 345–374, and EKD (C2-3) at 363–365. DV3-rED III showed six epitopes with QH (C3-1) at 315–316, DGQG (C3-2) at 340–343, A (C3-3) at 345, PFG (C3-4) at 371–373, Y (C3-5) at 391, and K (C3-6) at 393. DV4-rED III showed four epitopes with QH (C4-1) at 317–318, N (C4-2) at 344, G (C4-3) at 375, and K (C4-4) at 395. Position of each sequence is listed in Table 1.

### 2.3. Prediction of Position of Antigenic Epitope in Recombinant Antigens

**Figure 3:**
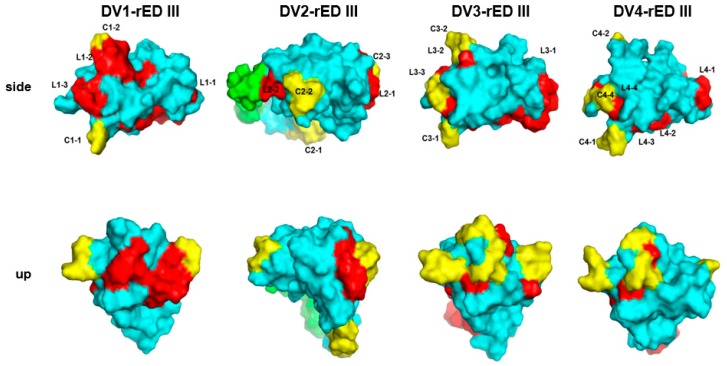
Characteristics of potential epitopes. Predicted common linear- and conformational epitopes of DV1-rED III to DV4-rED III were analyzed and presented after 3D modeling. Cyan color indicates the domain III of E protein, while green color indicates domain II. Red color indicates the common linear epitopes and yellow color represents the common conformational epitopes.

After each common epitope candidate was identified, location of the epitope was analyzed in the 3D structure of domain III of envelope protein, highlighted in red (linear epitope) and yellow (conformational epitope) in the 3D structure generated by I-tasser (Figure 3). In conformational epitope analysis, three well-known programs were applied to domain III of the four dengue type E proteins; ElliPro (threshold; 0.7), DiscoTope 2.0 (threshold; −3.7), and Bepro (threshold; 1.3), as previously described [26,27,28].

QH at 317–318 was commonly presented in three DV1 (C1-1), DV3 (C3-1), and DV4 (C4-1)-rED III as conformational epitopes. K (C1-2) at 344 was predicted at similar region in DGQG (C3-2) near 338–248 (Figure 3). In domain III of each type, the overall structure of three domains (DV1, DV3, and DV4) was similar although DV2-rED III had different structure.

The linear epitopes of DV1-rED III were mainly exposed to the outside compared to those of other types in 3D modeling. Those of DV2-, DV3-, and DV4-rED III were hidden, rather than being on the surface. The conformational epitopes of domain III of all four types of envelopes were commonly placed on the surface (Figure 3). Therefore, domain III seemed to share the very close epitopes across the serotypes in terms of both linear and conformational epitopes. However, DV1-domain III seems to present the strong epitopes on the surface more efficiently than other serotypes.

### 2.4. Clinical Cohort Study

Antigen-linked ELISA was used to evaluate serum samples from suspected dengue-infected patients provided by the National Children hospital in Vietnam between September and October 2017. A total of 22 patients were selected with acute dengue fever and were positive for NS1 with SD Rapid kit of Dengue NS1 when the specimens were collected.

There were more male than female patients, with an average age of 23.38 years. Specimens were collected within 2.6 ± 0.57 (mean ± SD) days after onset of fever. All suspected dengue-infected samples were collected at Ha Noi (*n* = 19), Bac Ninh (*n* = 1), Ha Nam (*n* = 1), and Hai Duong (*n* = 1), Vietnam (Table S1). The RT-PCR raw data of samples are shown in Figure S3.

Dengue type-specific RT-PCR and Zika virus-specific RT-PCR were conducted to confirm the patient’s disease. Out of 22 patient samples, 13 were from dengue type 1-infected patients and three were of dengue type 2. Five samples were negative in qRT-PCR and one sample might have co-infection with both Dengue type 1 and type 2 (Figure S3). All the samples were originally frozen at −80 °C for a year before ELISA.

As a negative control, healthy patients (*n* = 22), *Toxoplasma gondii*-infected patients (*n* = 10), *Plasmodium vivax*-infected patients (*n* = 10), and Zika virus-infected patients (*n* = 4) were used in this study.

### 2.5. DENV-Linked ELISA to Detect IgM in Clinical Samples

Each of the four recombinant antigens (DV1-, DV2-, DV3-, and DV4-rED III) was coated onto a 96-well plate independently, and patient sera (1:100 dilution) were applied to detect the antigen. Secondary antibody was used for the detection of patient IgM. Healthy individual samples (*n* = 22) are indicated as N, and *T. gondii*-infected samples (*n* = 10) as TG. *P. vivax*-infected samples (*n* = 10) are indicated as PV, and Zika virus-IgM positive sera (*n* = 4) are indicated as ZIKV. Dengue-positive samples are indicated as VN. The red dotted line indicates the cut-off value (mean + 3×SD).

### 2.6. Recombinant Antigen-Linked ELISA to Detect IgM in Clinical Samples

To determine the best domain III, among the four serotypes, to use as a diagnostic antigen, four different kinds of recombinant antigens—envelope protein domain III (rED III) derived from each type of dengue virus—were tested by ELISA. To determine the limits of antigen-based ELISA, different amounts of antigens (0.1, 0.5, 1, 5 µg/well) were used in 96-well plates under coating conditions.

Negative samples (*n* = 46) and positive samples (*n* = 22) were diluted in a blocking buffer of 5% BSA at a 1:100 ratio, as described previously [29].

The mean of the optical density of all negative samples (*n* = 46) plus three-fold standard deviation of all negative controls (Mean + 3 × SD) was designated as the cut-off point for distinguishing between positive and negative results, as described previously [30].

**Figure 4:**
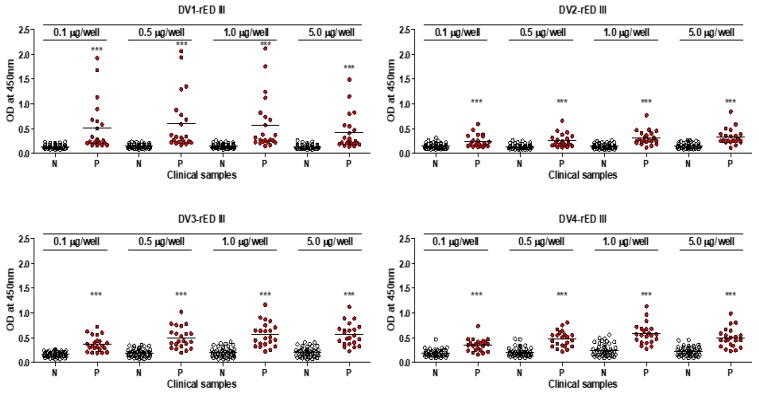
Clinical validation of antigen-linked ELISA with patient specimens. The dengue-negative sera, indicated as N (*n* = 46), and dengue-positive patient sera, indicated as P (*n* = 22), were applied in ELISA. Different amounts of antigens at 0.1, 0.5, 1, and 5 µg/well were tested with clinical samples and plotted in graph. ***, *p* < 0.001.

**Figure 5:**
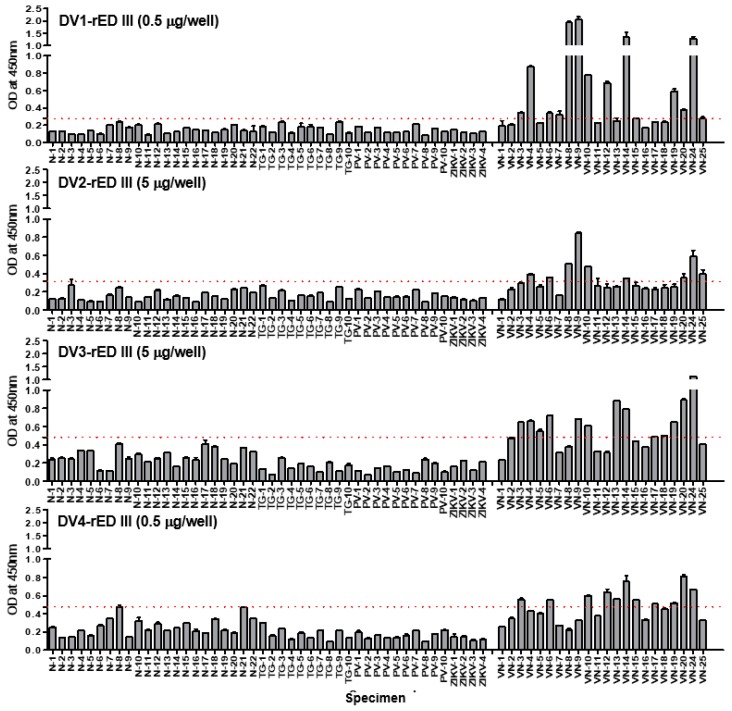
Recombinant antigen-linked ELISA with patient specimens.

The sensitivity of four single DV-rED III-based ELISA, at differential concentrations, is shown in Figure 4. Both DV1- and DV3-rED III showed 45.45–59.09% sensitivity in the dengue-positive sample, while DV2-rED III and DV4-rED III recognized only 27–40% and 31–50%, respectively (Table 2). Despite the increased antigen concentration from 0.1 to 5 µg/well, sensitivity of ELISA changed only slightly (13–19%). Under the condition of 0.5 µg/well, DV1-rED III showed the best sensitivity and highest OD values, with 0.277 as the cut-off value. It pointed out 12 samples (VN-3, -4, -6, -7, -8, -9, -10, -12, -14, -19, -20, and -24) as positive out of the 22 total samples, showing 59.09 % sensitivity (95% confidential interval (CI); 36.35 to 79.29%). In addition to sensitivity, DV1-rED III dramatically increased the OD value to 2. In contrast, three other dengue antigen-linked ELISA showed OD below 1, except for VN24 in DV3-rED III (Figure 4 and Figure 5).

Based on the cut-off value, the performance of ELISA had 100% specificity (46/46); and 92.29–100% for the 95% confidential interval (CI) (Table 2). Similarly, 5 µg/well DV2-rED III exhibited the highest sensitivity (40.91%, 9/22) at a cut-off value of 0.314.

Based on the cut-off values 0.483615 and 0.477119, DV3-rED III and DV4-rED III displayed the best sensitivity at 5 and 0.5 µg/well, with 59.09% (13/22) and 50% (11/22), respectively (Figure 5 and Table 2).

*T. gondii*-infected samples (*n* = 10), *P.vivax*-infected samples (*n* = 10) and Zika virus-IgM positive patients (*n* = 4) were all negative in recombinant antigen-based ELISA.

Four (VN-1, -2, -11, and -16) out of the 22 dengue-positive samples were negative for four antigens, and three of them showed high RNA content (Ct: 22.72–25.99), indicating that IgM level might be low due to the samples being in early acute phase (Table 3).

It was obvious that each antigen from DV1-, DV3, and DV4- rED III was able to compensate for the negative results derived from other antigen-linked ELISA (Table 3).

Table 4 supported this suggestion. In high viremia (20 < Ct ≤ 30), five out of eight samples were positive in DV-1, -3, and -4-rED III-ELISA, while three were negative for the antigen. However, in low viremia (30 < Ct ≤ 35), all samples (*n* = 4) were positive in ELISA. Likewise, five samples, all dengue type-negative in qRT-PCR, were positive for at least one of the antigens, indicating that when viremia is lower, IgM may be elevated.

Taken together, our study revealed that independent DV-1, -3, and -4-rED III-ELISA shows less than 60% sensitivity, although each antigen can compensate for the other antigen-derived negative results. The total combined results led to a sensitivity of 81.82% (18/22) (95% CI, 59.72 to 94.81) (Table 5). VN-1, VN-2, VN-11, and VN-16 were all negative for the whole dengue virus, implying that the samples’ IgM would be very low. Whole dengue virus-linked ELISA results was provided in Figure S4.

## 3. Discussion

Dengue is a major international public health concern, and is transmitted by DENV-carrying mosquitoes. Majority of dengue infections are asymptomatic cases, which causes difficulty in disease control and dengue surveillance [2,31]. Therefore, the availability of easy and reliable diagnostic methods can be useful for clinical surveillance and outbreak investigation. Since viremia occurs for only a short duration [32] (i.e., 1–2 days before onset of symptom and up to 5–7 days thereafter), the virologic/molecular/antigen-based tests are applicable in only symptomatic infectious cases, in which the disease onset has been noted [33]. However, due to high antigenic similarities with other flaviviruses, which co-circulate in dengue endemic areas, cross-reactive diagnostic methods hinder prompt arbovirus-infected patient management [34].

Diagnosis of acute (on-going) or recent dengue infection can be established by testing serum samples during the first 7 days of symptoms and/or early convalescent phase (more than 5 days of symptoms) [35]. Acute infections can also be confirmed in laboratory by identification of dengue viral antigen or RNA in autopsy tissue specimens. CDC recommends IgM antibody detection in patients who have IgM antibodies to dengue detected in their serum sample and had either a negative RT–PCR result in the acute phase sample or did not submit an acute phase sample; these are classified as having a recent probable dengue infection [35]. Recently, detection of the NS1 antigen during the acute phase of the disease in patients having primary and secondary infections was investigated in various laboratories across the world [36]. Currently, DENV NS1 rapid diagnostic test (RDT) showed about 88–99.2% sensitivity and 96.0–100% specificity when analyzed using DENV NS1 ELISA as the standard [37,38]. However, NS1 shows decreased sensitivity from 7 days after the onset of the illness [39] and cross-reactivity with Zika virus NS1 has commonly been reported [14]. Therefore, many efforts to find alternative diagnostic antigen has focused on the highly specific and immunogenic DENV envelope protein domain III (ED III) [24,40,41].

As ED III contains serotype-specific epitopes, it was recommended to utilize ED III (amino acid 296-416) derived from all serotypes of dengue by making chimeric tetravalent antigens; however, mixture of antigens or tetravalent antigens showed less than 63% sensitivity and 100% specificity [42]. In the current study, recombinant DENV ED III antigen from the four serotypes was expressed in *E. coli* host and when DV1;3;4-rED III results were combined, it showed great sensitivity (81.82%) at mainly 2–3 days after onset of fever.

Alternatively, DENV-1 EDIII expressed in *P. pastoris*, which is a more complicated and slower expression system than *E. coli*, showed comparable diagnostic performance (86.96% of sensitivity and 99.12% of specificity) after onset of fever [24].

In the beginning, we tried to use EDIII to differentiate between serotypes; however, the result was complicated by low specificity of each serotype, although their homologies were less than 66% (Figure S2). As a result, of epitope information, common epitopes (VNIEA or EPPFG in linear epitope, and QH in conformational epitope) were suggested as strong candidates in both linear and conformational epitope groups (repeatedly found in three independent serotypes), thus implying that domain III may be hardly serotype-specific. 3D modeling hinted at the strongest reactivity to patient IgM due to the most open position of linear epitopes on the surface. We consider this to cause the highest reactivity of DV1-rED III against patient and specificity, showing that two DV2 serotype samples (VN-5 and VN-18) were not detected with DV1-rED III.

According to the dramatically high value of OD in DV1-rED III, this antigen-linked ELISA could be optimized as a DV1-specific condition; however, in our setting, we could not conclude it due to lack of various serotypes in patients with dengue.

Differing influences of the virus burden and immune activation on disease severity of different type of DENV infections has been reported; DENV-1 or DENV-2 infections show higher circulating levels of replicating virus [10] or viral RNA copies [43] in DHF than in DF. Another study reported the increased severity of DENV-3 [9]. In this study, among DV1 serotype-specific samples (*n* = 13), 8 were positive in DV1-rED III while 5 were negative. We found that 4 out of 5 negative samples had high viremia (Ct below 30), which means that it may be too early to produce sufficient IgM. Among the four high-DV1 viremia samples, VN-1, -2, and VN-16 (Ct 22.72, 25.99, and 22.43) were all negative in four recombinant antigens, and VN-17 (Ct 35.76) was positive in only DV3- or DV4-rED III, and not in DV1-rED III. VN-15 (DV1 serotype and Ct 23.19) was positive in DV4-rED III only, hence implying that DV1-rED III should be tested with other serotype-derived recombinant antigens to compensate for the weakness of DV1-rED III.

Interestingly, two DV2 serotype-specific samples (VN-14 and VN-18) possessed Ct >30 and only VN-14 showed dramatically high OD value in DV1- and DV3-rEDIII. VN-18 was only positive in DV3-rED III. Therefore, our strategy could detect and help faster management of more than 80% of DV-1 and DV-2-infected patients using DV1-, DV3-, and DV4-rED III antigens, rather than by serotype-specific assay.

Five qRT-PCR negative dengue samples were wrongly detected as positive in at least one of the four whole-dengue serotype virus ELISA (see Figure S4). This may be attributed to the poor handling of specimens or the low viremia due to being in the late acute phase during sample collection.

Epitope analysis indicated that DV2- and DV4-rED III possess different epitopes compared with those of DV1- or DV3-rED III. Although DV2-rED III has the largest amino acid stretch over other type-derived antigen, its sensitivity was not high enough compared to DV1- or DV3-rED III. The epitope prediction results were compatible with ELISA results, indicating the important role of epitope analysis in diagnostic antigen quality.

DV2-rED III showed an inefficient diagnostic performance, implying that DV2-rED III might possess a low number of inefficient linear and conformational epitopes, which is consistent with the results of ELISA.

In 3D model structure, all three epitopes (DAP, FSTQDEKGATQ, and PVNIEAEPPFG) of DV1-rED III only were on the envelope protein surface, supporting the experimental result that DV1-rED III was more efficient than the other serotype rEDIIIs. Therefore, epitope prediction analysis by bioinformatics used in this study may be helpful to determine dengue-specific and serotype-specific antigens.

The limitation of this study was the small number of patients enrolled to evaluate the clinical performance of the antigens and lack of various dengue type-infected samples.

Further studies would be required to assess the quality of these recombinant antigens using a larger population and late-period samples (more than 3 days after onset of fever).

In conclusion, our newly designed recombinant antigens of DV1;3;4-ED were useful to diagnose IgM in patients with dengue just 2–4 days after disease onset.

## 4. Materials and Methods

### 4.1. Cell and Virus

*Aedes albopictus* clone C6/36 (ATCC CRL-1660TM) cells were purchased from American Type Culture Collection PO box 1549, Manassas, VA 20108 USA. The C6/36 cells were cultured in Dulbecco’s Modified Eagle Medium (DMEM), high glucose, pyruvate (Gibco) supplemented with 10% fetal bovine serum (FBS) (Gibco) and 1% antibiotic-antimycotic (Gibco) (Invitrogen, Carlsbad, CA, USA) at 28–30 °C with 5% CO2 in humidified incubator (Sanyo, Osaka, Japan).

The DENV strains used in this study were DENV-1 (Korea National Culture Collection for Pathogens - NCCP 41503; Genbank Accession: KP406803.1), DENV-2 (KBPV-VR-29; Genbank Accession: KP406804), DENV-3 (KBPV-VR-30; Genbank Accession: KP406805), and DENV-4 (KBPV-VR-31; Genbank Accession: KP406806).

### 4.2. Reagents

Taq polymerase enzyme and PCR reagents were purchased from Takara (Kyoto, Japan). Competent *E. coli* strain BL21 (DE3) and plasmid vector pET21b (+) were purchased from Novagen (Birmingham, UK). Restriction enzymes and T4 DNA ligase enzyme were purchased from NEB (New England Biolabs, Ipswich, MA, USA). HisPurTM Ni-NTA was purchased from Thermo Scientific (Meridian Rd, Rockford, IL, USA) and QIAGEN Plasmid Midi Kit was purchased from Qiagen (Qiagen GmbH, Hilden, Germany). Luria-Bertani broth, sodium chloride, urea, guanidine hydrochloride, L-arginine, and other chemicals were brought from Sigma Aldrich (St. Louis, MO, USA).

### 4.3. Expression of Recombinant Envelope Domain III Antigen

cDNA reverse transcribed from DENV RNA was used for PCR amplification of the domain III of each DENV-1, -2, -3, and -4 envelope protein (named as DV1-, DV2-, DV3-, DV4-rED III). Each of 304-bp DV1-rED III, 441-bp DV2-rED III, 304-bp DV3- rED III, and 286-bp DV4 rED III fragments was amplified by a pair of forward (F) and reverse primers (R): 5′-GGATCCGTCATATGTGATGTGC-3′ & 5′-CTCGAGTTTCTTGAACCAGCTTAGTTTC-3′; 5′-CCGGATCCCGCGCATCTTAAGTGCAGGCTGAGAATG-3′ & 5′-TTTAGCGGCCGCCCAGGCTGTGTCACCTAAAATGG-3′; 5′-GGATCCGAGCTATGCAATGT-3′ & 5′-CTCGAGTTTCTTATACCAGTTGATTTTC-3′; 5′-GGATCCGAAGTTCTCAATTGACAAAG-3′ & 5′-CTCGAGCCCTTTCCTGAACCAAT-3′, respectively. The PCR thermo cycling parameters were as follows: initial denaturation at 95°C for 5 min; 30 cycles of denaturation at 94 °C for 30 s, annealing at 55 °C for 45 s and elongation at 68 °C for 45 s; followed by final extension at 68 °C for 7 min. PCR amplicons were cloned in pET21b (+) plasmid vector and induced with 0.5 mM isopropyl-b-D-thiogalactopyranoside (IPTG) (Sigma). All the proteins in pellet were solubilized and refolded as described previously [44].

The recombinant His-tagged proteins were purified using HisPurTM Ni-NTA resin according to the manufacturer’s instruction, and concentrated with Centricon filter unit. Protein expression was examined by western blot analysis. Briefly, the purified proteins were loaded onto 15% SDS-PAGE gel, and then transferred to nitrocellulose membranes. The membrane was blocked with blocking buffer (5% non-fat milk in PBS) for 2 h at RT. After washing with PBS containing 0.1% Tween 20 (PBS-T), the membranes were incubated with the first anti-mouse 6×His-tag antibody (dilution 1:5000) in PBS-T containing 5% BSA for overnight at 4 °C. Following three washes with PBS-T, the membranes were incubated with the secondary antibody anti-mouse conjugated with HRP (Abcam), diluted (1:30000) in blocking buffer for 45 min at RT. After three washes with PBS-T, the protein bands were visualized using Bio-Rad ChemiDoc XRS+ (Hercules, CA, USA).

### 4.4. RT-PCR

Patient serum samples were screened using RT-PCR to determine the dengue type. Ct values ≤36 were considered as positive samples [45]. The Ct values are shown in Table 4. Briefly, viral RNA was isolated from 200 µL of each patient serum and from dengue virus-infected C6/36 cell supernatant as positive sample, using QIAamp viral RNA Kit (Qiagen, Germany) according to the manufacturer’s protocol. Real-time RT-PCR (Taqman) assay was performed in a total volume of 25 µL One-Step RT-PCR kit (Bio-Rad, CA) using 5 µL RNA as template, 10 µM probe, and 10 µM of each serotype-specific primer (probe and primer sequences were reported in a previous study) [46]. Reverse transcription at 50 °C for 30 min was followed by a cycle of initial denaturation (95 °C/1 min), and 40 subsequent cycles of denaturation (94 °C/15 s) and primer annealing (60 °C/30 s). All samples were screened for qRT-PCR using Zika virus-specific probe to confirm, as previously described [47].

### 4.5. ELISA

To determine the IgM level of each patient, the complete dengue virus was tested by indirect enzyme-linked immunosorbent assay (ELISA). Briefly, different dengue recombinant envelope protein domain IIIs (rED III) antigens, derived from the four DENV serotypes, were coated on the bottom of 96-well polystyrene plate (NUNC, Pasadena, TX, USA) in 50 mM of bicarbonate buffer pH 9.6 at 4 °C overnight. The plate was washed with PBS-T and then blocked with 5% non-fat milk at 37 °C. After washing, human sera patient samples diluted (1:100) in blocking buffer were added to each well and incubated at 37 °C for 3 h. The samples were then washed and incubated with secondary antibody HRP-conjugated anti-human IgM, according to the manufacturer’s protocol. Stringent washing with PBS-T was performed five times to remove all nonspecific binding and 3,3′5,5′-tetra methyl benzidine (TMB) substrate solution (Invitrogen) was added to each well and the plates were kept in dark for 15 min. Finally, the reaction was stopped by 0.18 M sulfuric acid. Optical density (OD) was determined using the SpectraMax® M Series Multi-Mode Microplate Readers (Molecular Devices, San Jose, CA 95134, USA) at 450 nm.

### 4.6. ED III Sequence Analysis and Protein Structure Graphic

To analyze the potential linear epitopes, ABCpred, BCPreds server 1.0, and IEDB-BepiPred were used as described previously [47,48,49,50]. To analyze the conformational epitope, Discotope, BEPro, and Ellipro were used as previously reported [26,51,52]. The three-dimensional structure (3D) was prepared using I-TASSER for each of the four serotypes of dengue ED III, and visualized using the molecular graphic program PyMOL (http://www.pymol.org) [47].

### 4.7. Ethics Statement

Blood samples from patients with dengue (*n* = 22) were collected from endemic areas of Vietnam, and healthy individual samples (dengue-negative sera) (*n* = 22) were collected from a non-endemic area of malaria (Iksan province, Korea) and tested for dengue and Zika infection by rRT-PCR. *T. gondii*-infected samples (*n* = 10) and *P. vivax*-infected samples (*n* = 10) were prepared as previously described [53]. Four Zika-IgM positive sera (*n* = 4) were purchased from ABO Pharmaceuticals (San Diego, CA, USA) as additional negative sera. All study participants provided informed consents before providing specimens. The study was approved by the Wonkwang University Hospital Institutional Review Board (Approval No. 201603-BR-015). The dengue-positive sera were tested by NS1 antigen kit (SD BIOLINE, Lake Bluff, IL, USA) following manufacture’s instruction.

### 4.8. Statistical Analysis

All graphs were generated using GraphPad Prism (Version 5.0, La Jolla, CA, USA). One-way analysis of variance (ANOVA) was used for analyzing ELISA. A *p* value < 0.05 was considered as statistically significant.

## Figures and Tables

**Table 1:** Potential linear and conformational epitopes predicted by three independent tools.

Dengue Type	Linear		Conformational	
DV1-rED III	L1-1 L1-2 L1-3	DAP (331-333) ^a^ FSTQDEKGATQ (337-347) PVNIEAEPPFG (365-375)	C1-1 C1-2	QH (317-318) K (344)
DV2-rED III	L2-1 L2-2	EGDGSP (330-335) TEKDRPVNIEAEPPFG (362-377)	C2-1 C2-2 C2-3	RLRMDKLQ (289-296) LEK (345-347) EKD (363-365)
DV3-rED III	L3-1 L3-2 L3-3	KGEDAP (326-331) VVTKKEEPVNIEAEPP (356-371) E (374)	C3-1 C3-2 C3-3 C3-4 C3-5 C3-6	QH (315-316) DGQG (340-343) A (345) PFG (371-373) Y (391) K (393)
DV4-rED III	L4-1 L4-2 L4-3 L4-4	EGAGAP (328-333) N (367) E (369) EPPFG (371-375)	C4-1 C4-2 C4-3 C4-4	QH (317-318) N (344) G (375) K (395)

^a^ Position of amino acid.

**Table 2:** Sensitivity and specificity of each antigen-linked ELISA.

Antigens	µg/well	Cut-off	Sensitivity (*n* = 22)		Specificity (*n* = 46)	
			% (Positive No.)	95% CI ^a^	% (Negative No.)	95% CI
DV1-rED III	**0.1**	0.269527	45.45 (10)	24.39 to 67.79	100.00 (46)	92.29 to 100.00
	**0.5 ^b^**	**0.276739**	**59.09 (13)**	**36.35 to 79.29**	**100.00 (46)**	**92.29 to 100.00**
	1	0.286781	50.00 (11)	28.22 to 71.78	100.00 (46)	92.29 to 100.00
	5	0.270051	45.45 (10)	24.39 to 67.79	100.00 (46)	92.29 to 100.00
DV2-rED III	0.1	0.314	27.27 (6)	10.73 to 50.22	100.00 (46)	92.29 to 100.00
	0.5	0.282	31.82 (7)	13.86 to 54.87	100.00 (46)	92.29 to 100.00
	1	0.295	40.91 (9)	20.71 to 63.65	100.00 (46)	92.29 to 100.00
	**5**	**0.314**	**40.91 (9)**	**20.71 to 63.65**	**100.00 (46)**	**92.29 to 100.00**
DV3-rED III	0.1	0.324308	54.55 (12)	32.21 to 75.61	100.00 (46)	92.29 to 100.00
	0.5	0.434963	45.45 (10)	24.39 to 67.79	100.00 (46)	92.29 to 100.00
	1	0.481123	54.55 (12)	32.21 to 75.61	100.00 (46)	92.29 to 100.00
	**5**	**0.483615**	**59.09 (13)**	**36.35 to 79.29**	**100.00 (46)**	**92.29 to 100.00**
DV4-rED III	0.1	0.402995	31.82 (7)	13.86 to 54.87	100.00 (46)	92.29 to 100.00
	**0.5**	**0.477119**	**50.00 (11)**	**28.22 to 71.78**	**100.00 (46)**	**92.29 to 100.00**
	1	0.606018	36.36 (8)	17.20 to 59.34	100.00 (46)	92.29 to 100.00
	5	0.492167	45.45 (10)	24.39 to 67.79	100.00 (46)	92.29 to 100.00

^a^ CI; Confidence interval. ^b^ bold; the most efficient antigen concentration.

**Table 3:** Diagnostic performance of antigen-linked ELISA using recombinant antigens under best condition.

Patients	Days after Onset of Disease	RT-PCR (Dengue Type/Ct)	RT-PCR (Zika Virus/Ct)	ELISA			
				DV1-rED III (0.5 µg/well)	DV2-rED III (5 µg/well)	DV3-rED III (5 µg/well)	DV4-rED III (0.5 µg/well)
VN-1	3	1(22.72)	N	N	N	N	N
VN-2	3	1(25.99)	N	N	N	N	N
VN-3	2	1(29.15)	N	P ^b^	N	P	P
VN-4	2	1(30.15)	N	P	P	P	N
VN-5	3	2(35.03)	N	N	N	P	N
VN-6	4	1(27.06)	N	P	P	P	P
VN-7	3	1(35.66)	N	P	N	N	N
VN-8	2	N ^a^	N	P	P	N	N
VN-9	3	N	N	P	P	P	N
VN-10	3	1(25.17)	N	P	P	P	P
VN-11	3	1(35.57)/2(35.89)	N	N	N	N	N
VN-12	2	N	N	P	N	N	P
VN-13	3	N	N	N	N	P	P
VN-14	2	2(33.83)	N	P	P	P	P
VN-15	2	1(23.19)	N	N	N	N	P
VN-16	2	1(22.43)	N	N	N	N	N
VN-17	3	1(35.76)	N	N	N	P	P
VN-18	2	2(35.50)	N	N	N	P	N
VN-19	3	1(31.50)	N	P	N	P	P
VN-20	2	1(24.64)	N	P	P	P	P
VN-24	3	1(33.31)	N	P	P	P	P
VN-25	3	N	N	P	P	N	N

^a^ Negative. ^b^ Positive.

**Table 4:** Diagnostic performance of DV1-rED III-linked ELISA at different times after the onset of disease.

rRT-PCR (Dengue)	ELISA (DV1-rED III) + ELISA (DV3-rED III) + ELISA (DV4-rED III)						Total (*n* = 22)	
	2 Days after Onset of Disease		3 Days after Onset of Disease		4 Days after Onset of Disease			
							Neg.	Pos.
	Negative	Positive	Negative	Positive	Negative	Positive		
20 < Ct ≤ 30 (*n* = 8)	1 (VN16 ^1,2,3,4^)	3 (VN3 ^1,3,4^; VN15 ^4^; VN20 ^1,2,3,4^)	2 (VN1 ^1,2,3,4^; VN2 ^1,2,3,4^)	1 (VN10 ^1,2,3,4^)		1 (VN6 ^1,2,3,4^)	3	5
30 < Ct ≤ 35 (*n* = 4)		2 (VN4 ^1,2,3^; VN14 ^1,2,3,4^)		2 (VN19 ^1,3,4^; VN24 ^1,2,3,4^)				4
35 < Ct (*n* = 5)		1 (VN18 ^3^)	1 (VN11 ^1,2,3,4^)	3 (VN5 ^3^; VN7 ^1^; VN17 ^3,4^)			1	4
Negative (*n* = 5)		2 (VN8 ^1,2^; VN12 ^1,4^)		3 (VN9 ^1,2,3^; VN13 ^3,4^; VN25 ^1,2^)				5
Total (*n* = 22)	1	8	3	9		1	4	18

^1,2,3,4^ Negative or Positive by DV1;2;3;4-rED III-based ELISA.

**Table 5:** Combined result of DV-rED III-ELISA sensitivity and specificity.

Parameter	DV1-rED III	DV2-rED III	DV3-rED III	DV4-rED III	Combined Result of DV1-, DV3-, and DV4-rED III
Sensitivity% (95% CI)	59.09 (13/22) (36.35 to 79.29)	40.91 (9/22) (20.71 to 63.65)	59.09 (13/22) (36.35 to 79.29)	50.00 (11/22) (28.22 to 71.78)	81.82 (18/22) (59.72 to 94.81)
Specificity% (95% CI)	100.00 (46/46) (92.29 to 100.00)	100.00 (46/46) (92.29 to 100.00)	100.00 (46/46) (92.29 to 100.00)	100.00 (46/46) (92.29 to 100.00)	100.00 (46/46) (92.29 to 100.00)

## Supplementary Materials

Supplementary materials can be found at https://www.mdpi.com/1422-0067/20/14/3464/s1.
