# Supplementary material for: Diagnostic Performance of Dengue Virus Envelope Domain III in Acute Dengue Infection
Source: Int J Mol Sci. 2019 Jul 15;20(14):3464. doi: 10.3390/ijms20143464 (PMC6679088; doi:10.3390/ijms20143464)
Supplement: Supplementary file 1 [file ijms-20-03464-s001.pdf]

Supplementary Information

Diagnostic Performance of Dengue Virus Envelope Domain IIIs of Acute Dengue Infection

1. Method

Dengue virus propagation and titration

2. Result

Figure S1. Titration of Dengue virus.

Figure S2. Multiple sequence alignment of ED IIIs derived from four dengue virus envelope.

Figure S3. RT-PCR of Dengue-Positive samples.

Figure S4. Whole dengue-virus liked ELISA of patient samples.

Table S1. Characteristics of suspected dengue-infected patients.

Dengue Virus Propagation and Titration

C6/36 cells were cultured until 70–90% confluence, and then infected with DENV with multiplicity of infection (M. O. I) = 0.1 in DMEM without FBS. Virus adsorption was conducted at 28–30 °C, 5% CO_2 for 2–3 h with occasional mixing at 20 min intervals. Then, the virus mixture was removed and maintenance media (DMEM), supplemented with 2% FBS, was added to the flask, which was incubated at 37 °C and 5% CO_2. After 7 days of incubation, the medium containing the virus supernatant was harvested from the cells and clarified by centrifugation at 4000× *g* for 30 min. Fresh maintenance media supplemented with 2% FBS was repeatedly added to the cell culture. The virus supernatant was harvested at 14 and 21 days post infection. The supernatant was filtered through a 0.45-µm filter (GVS filter technology, Sanford, ME, USA), followed by sucrose ultracentrifugation [1]. Titration of virus was obtained using focus forming assay (FFU) conducted as previously reported [2]. Briefly, virus supernatants collected from C6/36 cell culture were serially 10-fold diluted in DMEM and then transferred to monolayers of Vero cells in 24-well microplates. After incubation for 2 h at 37 °C with occasional mixing, the infected cells were further incubated with 1.25% carboxylmethyl-cellulose (CMC) (Sigma, Saint Louis, MO, USA) in 2% FBS-DMEM for 72 h at 37 °C, 5% CO_2 in incubator. The plates were washed thrice with PBS to remove CMC, then fixed with a 3.7% para-formaldehyde solution, and permeabilized by treatment with 0.1% Triton X-100 prior to blocking with buffer containing 3% BSA in PBS, 0.1% Tween 20, and 22.52 mg/mL glycine. The samples were then incubated with the primary antibody anti-*flavivirus* group antigen 4G2 (Merck, Darmstadt, GE,) for 2 h at room temperature (RT), followed by incubation with the secondary antibody, horseradish peroxidase (HRP)-conjugated rapid anti-mouse IgG (H&L) (Abcam, Cambridge, UK) for 1 h at RT. The culture plates were continuously washed with PBS five times and the infected cells were visualized by reaction with H_2O_2–diaminobenzidine (Sigma, Saint Louis, MO, USA) and foci were counted.

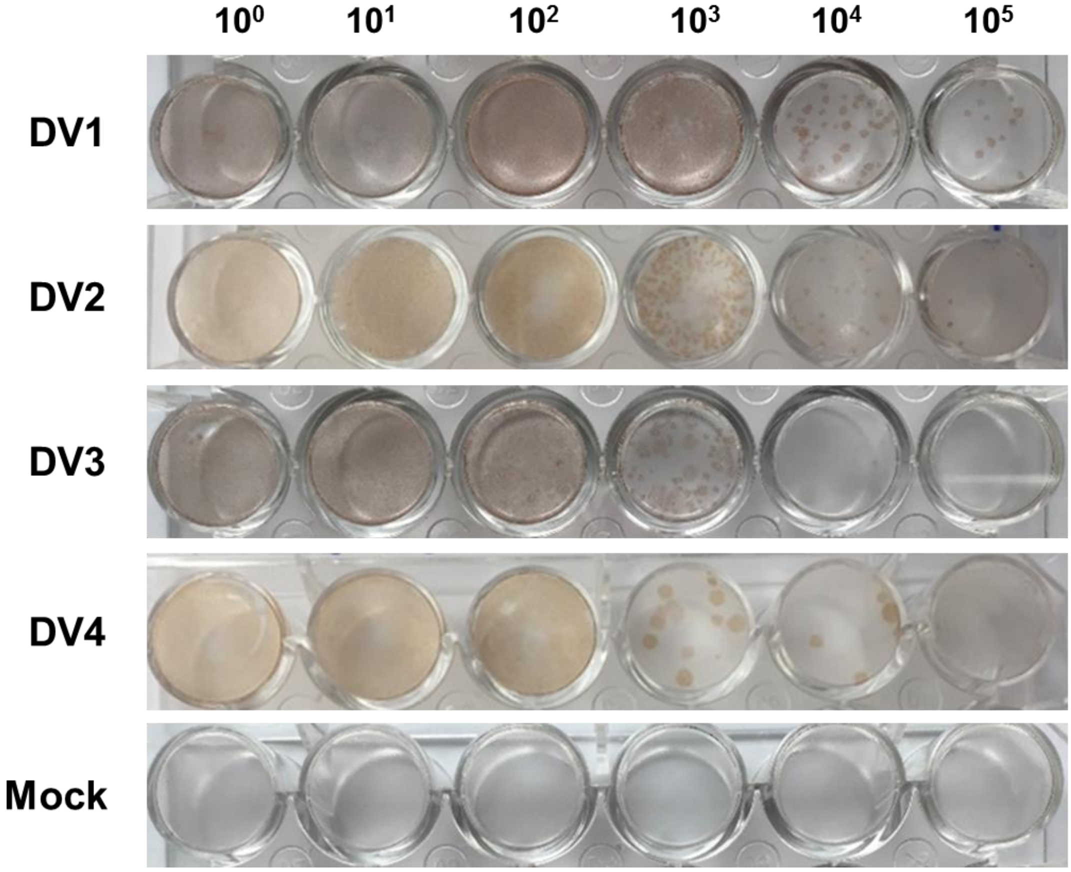

Figure S1. Titration of Dengue virus. Foci formation of DV-1;2;3;4.

The titer of DV-1;2;3;4 was determined by focus forming assay in mosquito C6/36 cell line. The brown foci were indicative of dengue virus. Vero cell monolayers were infected with four different serotypes of dengue virus (200 µL/well) and mock with viral dilution medium (DMEM) (200 µL/well) then covered with a CMC overlay. The cultures were fixed and incubated with anti-flavivirus group antigen 4G2 (NativeAntigen, Oxford, UK), followed by horseradish peroxidase (HRP)-conjugated rapid anti-mouse IgG (H&L). The foci were visualized by reacting the samples with H_2O_2–DAB. The titer of DV-1;2;3;4 were 6×10^6, 2×10^6; 5×10^4; 25×10^4 ffu/mL, respectively.

Analysis of rED III SEQUENCE

To determine the percentage identity between the four recombinant antigens, the a.a. sequences were compared and analyzed (Figure S2a). DENV-1 and DV3-rED IIIs showed high homology (66%/87% identities/positives) compared with those of DV2-or DV4 rED III. Alignment results of the four different rED IIIs are shown in Figure S2b.

a

	DV1-rED III (Identities/Positives)	DV2-rED III (Identities/Positives)	DV3-rED III (Identities/Positives)	DV4-rED III (Identities/Positives)
DV1 ED III	100%			
DV2 ED III	62% (79%)	100%		
DV3 ED III	66% (87%)	56% (77%)	100%	
DV4 ED III	51% (68%)	58% (72%)	47% (68%)	100%

b

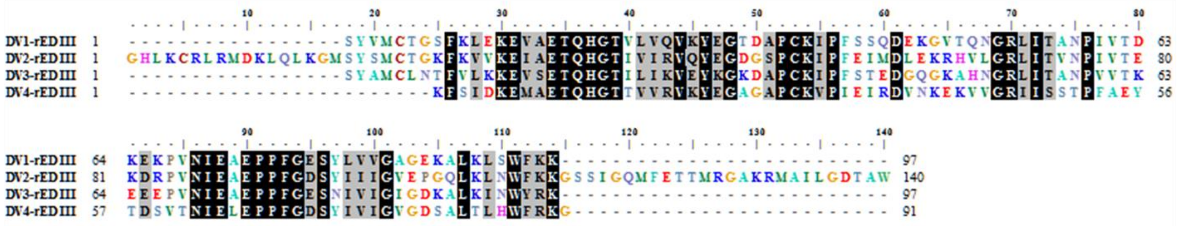

Figure S2. Multiple sequence alignment of ED IIIs derived from four dengue virus envelopes. (**a**) Amino acid (a.a.) sequences of four recombinant antigens (DV1-rED III, DV2-rED III, DV3-rED III, DV4-rED III) were aligned in BLAST, using the BLASTP 2.2.29+ program. The a.a. identity is summarized. (**b**) Four antigens derived from different DENV serotypes were aligned and analyzed by BioEdit. Identical residues are shaded in black, while similar amino acids are shown in gray background.

Table S1. Characteristics of suspected dengue-infected patients.

**Patients**	**Gender**	**Year of Birth**	**Days after Onset of Disease**	**Date of Sample Collection**	**Location**	**RT-PCR (Dengue)**	RT-PCR (Zika)
VN-3	Male	2005	2	17-Oct-17	Ha Noi	1	-
VN-4	Female	1982	2	17-Oct-17	Bac Ninh	1	-
VN-9	Female	1954	2	17-Oct-17	Ha Noi	Uk[a]	-
VN-12	Female	1989	2	17-Oct-17	Hai Duong	Uk	-
VN-14	Male	2013	2	17-Oct-17	Ha Noi	2	-
VN-15	Female	1977	2	18-Oct-17	Ha Noi	1	-
VN-16	Male	1996	2	18-Oct-17	Ha Noi	1	-
VN-18	Female	1999	2	18-Oct-17	Ha Noi	2	-
VN-20	Male	1996	2	18-Oct-17	Ha Noi	1	-
VN-1	Female	1965	3	17-Oct-17	Ha Noi	1	-
VN-2	Male	1996	3	17-Oct-17	Ha Noi	1	-
VN-5	Female	1979	3	17-Oct-17	Ha Noi	2	-
VN-7	Male	1989	3	17-Oct-17	Ha Noi	1	-
VN-8	Male	1979	3	17-Oct-17	Ha Nam	Uk	-
VN-10	Female	1997	3	17-Oct-17	Ha Noi	1	-
VN-11	Male	1949	3	17-Oct-17	Ha Noi	1/2	-
VN-13	Female	1996	3	17-Oct-17	Ha Noi	Uk	-
VN-17	Male	1998	3	18-Oct-17	Ha Noi	1	-
VN-19	Male	1998	3	18-Oct-17	Ha Noi	1	-
VN-24	Male	2012	3	25-Nov-17	Ha Noi	1	-
VN-25	Female	2014	3	25-Nov-17	Ha Noi	Uk	-
VN-6	Male	2006	4	17-Oct-17	Ha Noi	1	-

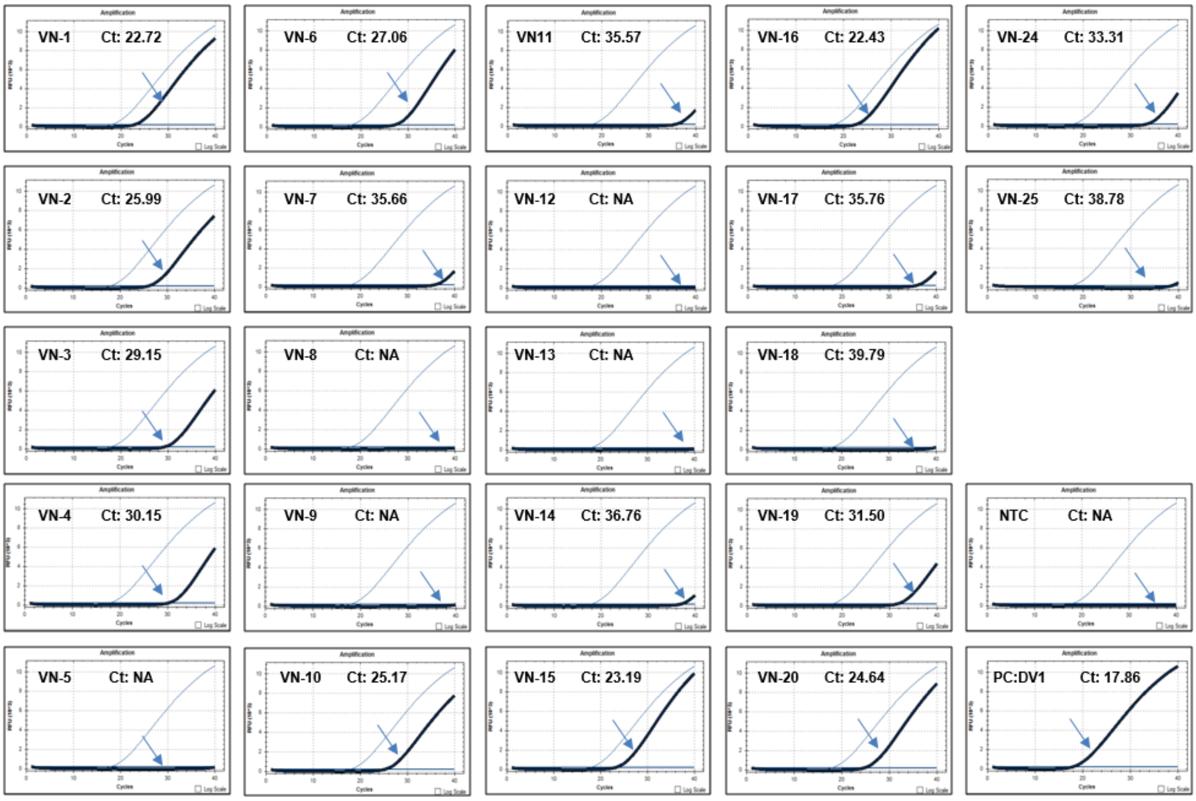

(A) Dengue type 1 probe.

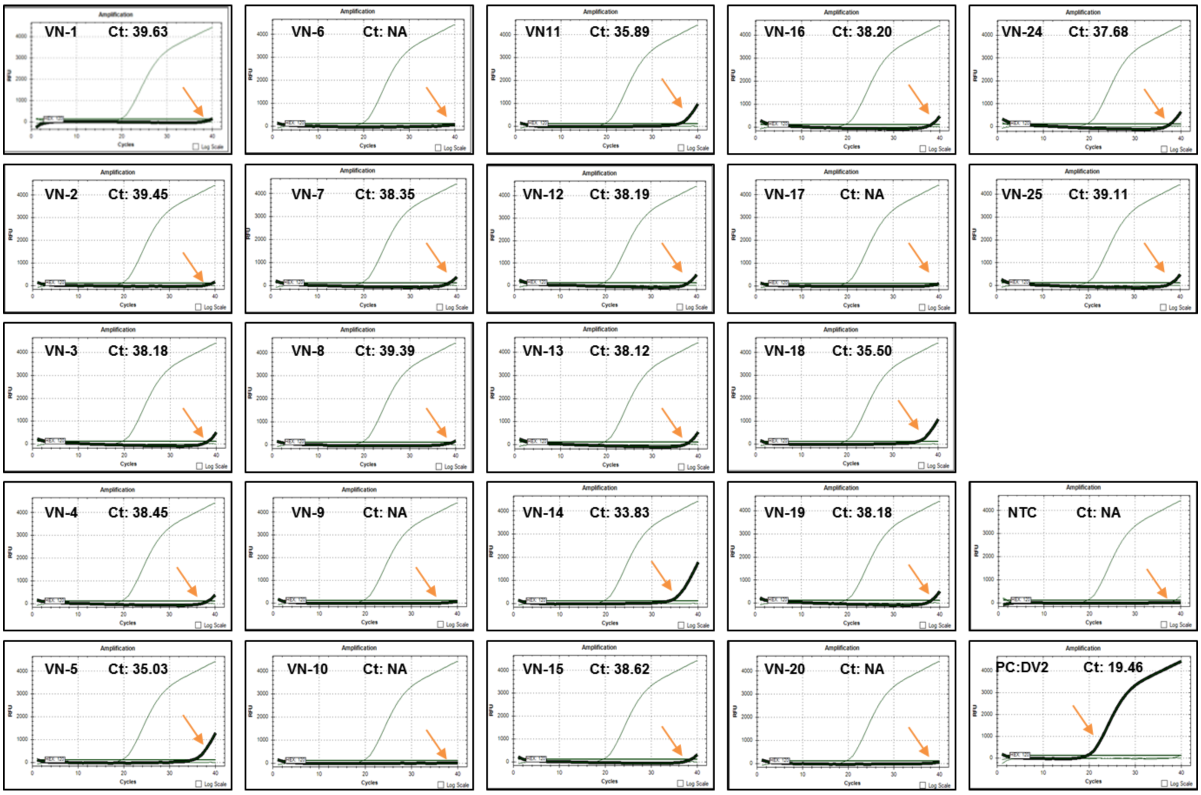

(B) Dengue type 2 probe

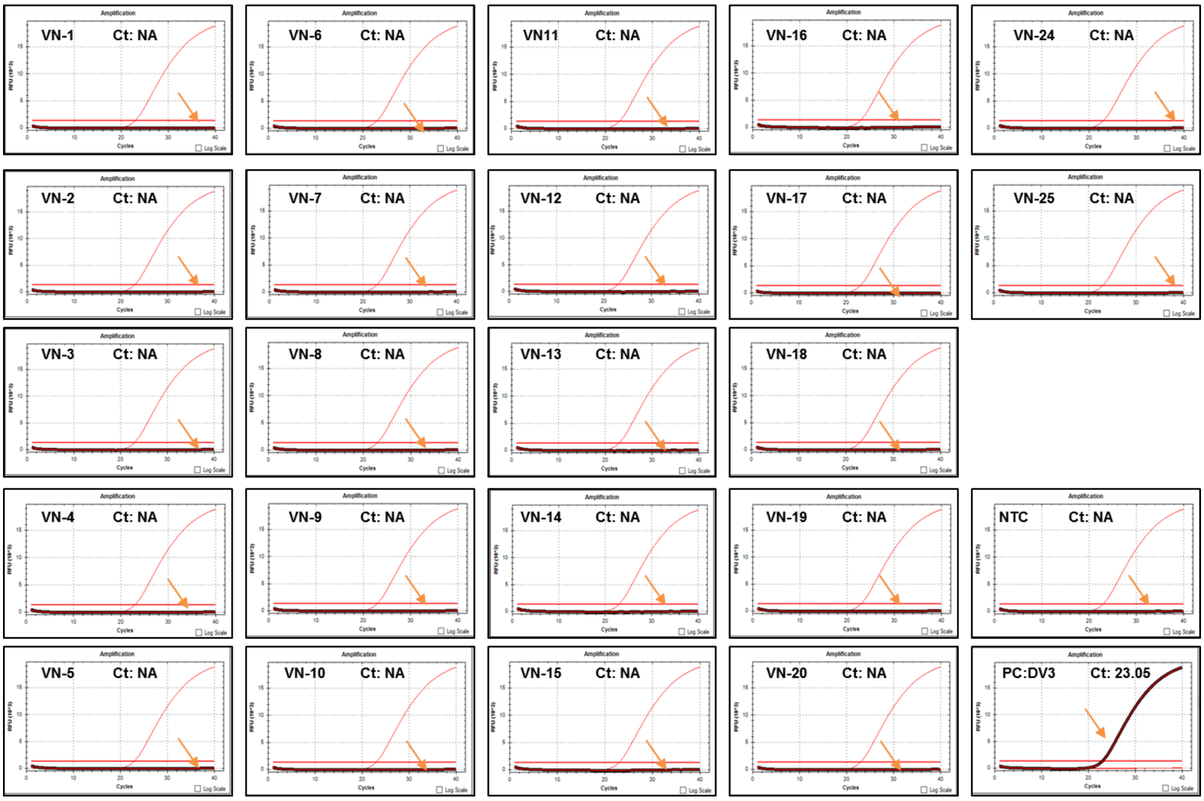

(C) Dengue type 3probe

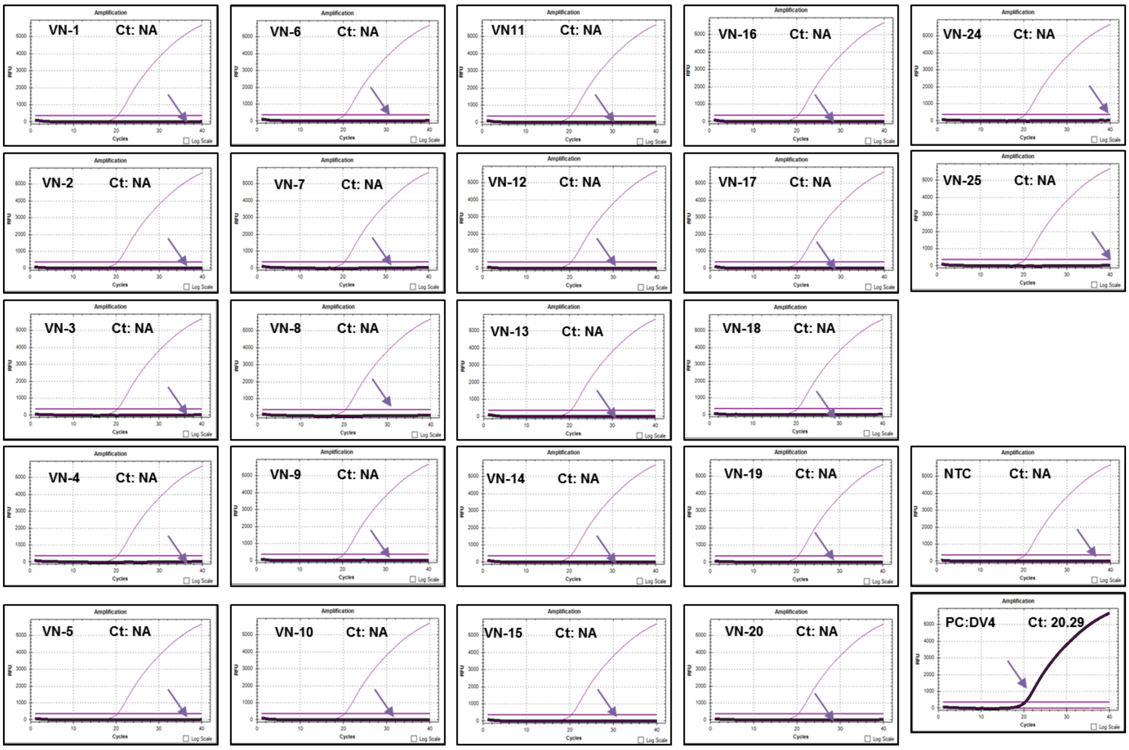

(D) Dengue type 4 probe

Figure S3. RT-PCR of dengue-positive samples.

(**A**) DV1-specific rRT-PCR. (**B**) DV2-specific rRT-PCR. (**C**) DV3-specific rRT-PCR. (**D**) DV4-specific rRT-PCR. NTC, non-templated control; PC, positive control.

(A)

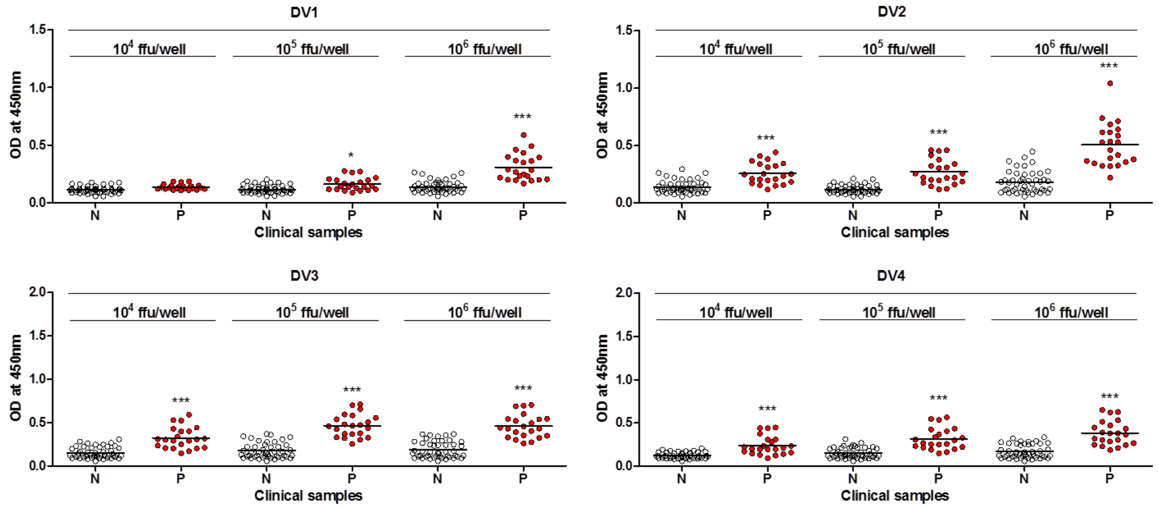

(B)

Patients	Days after onset of disease	RT-PCR (Dengue type/ Ct)	RT-PCR (Zika virus/ Ct)	ELISA							
				DV1		DV2		DV3		DV4	
				Whole virus	rED III (0.5 μg/well)	Whole virus	rED III (0.5 μg/well)	Whole virus	rED III (5 μg/well)	Whole virus	rED III (5 μg/well)
**VN-1**	3	1(22.72)	N	N	N	N	N	N	N	N	N
**VN-2**	3	1(25.99)	N	N	P	N	P	N	P	N	P
**VN-3**	2	1(29.15)	N	N	P	N	P	P	P	N	P
**VN-4**	2	1(30.15)	N	N	P	N	P	N	P	N	P
**VN-5**	3	2(35.03)	N	N	P	N	N	P	P	N	P
**VN-6**	4	1(27.06)	N	P	P	P	P	P	P	P	P
**VN-7**	3	1(35.66)	N	N	P	N	N	N	N	N	N
**VN-8**	2	N	N	N	P	P	P	N	P	N	N
**VN-9**	3	N	N	N	P	P	P	P	P	N	N
**VN-10**	3	1(25.17)	N	P	P	P	P	P	P	P	P
**VN-11**	3	1(35.57)/2(35.89)	N	N	P	N	N	N	N	N	N
**VN-12**	2	N	N	P	P	P	P	P	N	P	P
**VN-13**	3	N	N	N	P	N	P	P	P	P	P
**VN-14**	2	2(33.83)	N	P	P	P	P	P	P	N	P
**VN-15**	2	1(23.19)	N	N	P	N	P	N	P	N	P
**VN-16**	2	1(22.43)	N	N	N	N	P	N	P	N	N
**VN-17**	3	1(35.76)	N	P	P	P	P	P	P	P	P
**VN-18**	2	2(35.50)	N	P	P	N	P	P	P	P	P
**VN-19**	3	1(31.50)	N	P	P	P	P	P	P	P	P
**VN-20**	2	1(24.64)	N	P	P	P	P	P	P	P	P
**VN-24**	3	1(33.31)	N	P	P	P	P	P	P	P	P
**VN-25**	3	N	N	P	P	P	P	N	P	N	P

Figure S4. Whole dengue virus-linked ELISA.

(**A**) All specimens were tested in different dengue virus amounts at 10^4, 10^5, and 10^6 ffu/well. *, P <0.05; ***, P < 0.001. N, dengue-negative patient; P, dengue-positive patient. (**B**) Both recombinant antigens and whole dengue virus-linked ELISA data are summarized. P, positive result; N, negative result.

Reference

1. Gromowski, G.D.; Barrett, N.D.; Barrett, A.D. Characterization of dengue virus complex-specific neutralizing epitopes on envelope protein domain III of dengue 2 virus. *J. Virol.* **2008**, *82*, 8828–8837.
2. Kurosu T, Khamlert C, Phanthanawiboon S, Ikuta K, Anantapreecha S. Highly efficient rescue of dengue virus using a co-culture system with mosquito/mammalian cells. *Biochem. Biophys Res. Commun.* 2010, 394, 398–404.
